# Extra‐Amniotic Bovine Foetus With Aplasia of the Posterior Body: A Case Report

**DOI:** 10.1111/rda.70137

**Published:** 2025-10-21

**Authors:** Luís Filipe Moreira Martins Esteves, Juhani Taponen

**Affiliations:** ^1^ Department of Production Animal Medicine University of Helsinki Saarentaus Finland

**Keywords:** congenital defects, extra amniotic pregnancy, ultrasound

## Abstract

To date, extra‐amniotic foetal development has not been reported in cattle. In humans, such development outside the amniotic cavity is often associated with the formation of amniotic strands, which can lead to developmental malformations. Although congenital defects in the bovine species are uncommon, they typically result in significant economic losses due to production losses and additional veterinary costs. In this case, pregnancy was diagnosed in a primiparous Jersey‐Holstein crossbred cow, 56 days after insemination, during routine pregnancy evaluation. Transrectal palpation revealed less uterine enlargement (i.e., corresponding to a 6‐week pregnancy) than was expected for the gestational age. Ultrasound examination (using a Draminski, Poland) confirmed a right‐horn pregnancy with an ipsilateral corpus luteum. Foetal length was measured at 41 mm, compared to the expected 50 mm at 56 days of gestation, according to the ultrasound software. The foetus exhibited severe malformations, including the complete absence of limbs and gross deformities caudal to the neck. Although the amnion was fluid‐filled, the foetus was located outside of it, within the allantochorion. Abortion was induced, but the aborted material was not recovered. The cow successfully conceived again after one oestrous cycle and calved without complications. While most congenital defects are diagnosed postpartum, this case underscores the importance of performing routine ultrasound pregnancy diagnosis to enable the early detection of developmental abnormalities, facilitating timely intervention and reducing economic losses. To our knowledge, this is the first documented case of an extra‐amniotic pregnancy in cattle.

## 1 Introduction

Extra‐amniotic pregnancies are defined as the development of a foetus wholly or partly outside the amniotic sac. This occurs due to the preterm rupture of the amnion and in the absence of amniotic band repercussions (Castro and McKay 2018). However, this has not been documented in cattle.

In humans, amnion rupture sequence has been associated with amniotic strands surrounding the foetus (Gică et al. 2024). These fibrous strands can constrict the extremities and impair blood flow, leading to necrosis and intrauterine amputations. This is usually known as amniotic band syndrome (or sequence), and its consequences vary greatly (e.g., limb or digit amputation, or facial deformities) depending on the timing and location of the formed bands (Bhui et al. 1983; Gandhi et al. 2019).

In cattle, congenital abnormalities are rare and are mostly noticed around calving. In Ireland, during the year of 2023, “Hereditary and developmental abnormality” was diagnosed post‐mortem in 4.9% of neonatal calves, and in 1.2% in calves aged between 1 and 6 months (Department of Agriculture, Food and the Marine of Ireland et al. 2024). The total absence of at least one limb (i.e., amelia) is even rarer. In two Irish studies, 1 and 4 cases were reported among 191 and 522 cases of congenital abnormalities, respectively. In both cases, they represent less than 1% of the reported congenital defects (Mee et al. 2023; Quigley and Mee 2025).

Given that reports on extra‐amniotic bovine foetal development are non‐existent, and those on large congenital defects are scarce, we report a case of extra‐amniotic pregnancy with a large body defect that was identified during routine pregnancy diagnosis at a dairy farm.

## 2 Ethics Statement

This case report describes the clinical findings observed during routine veterinary practice. Ethical approval was not required, as no experimental procedures were performed. All diagnostic and treatment procedures were done according to standard veterinary care and welfare guidelines.

## 3 Case Presentation

A primiparous Jersey‐Holstein crossbred dairy cow was being examined for pregnancy diagnosis 56 days after being inseminated. During transrectal palpation, the animal was diagnosed with a right‐horn pregnancy, and a corpus luteum was identified on the right ovary. However, the uterus size did not seem to correspond to the expected length of pregnancy, estimated as the size of a 6‐week pregnancy.

**FIGURE 1:**
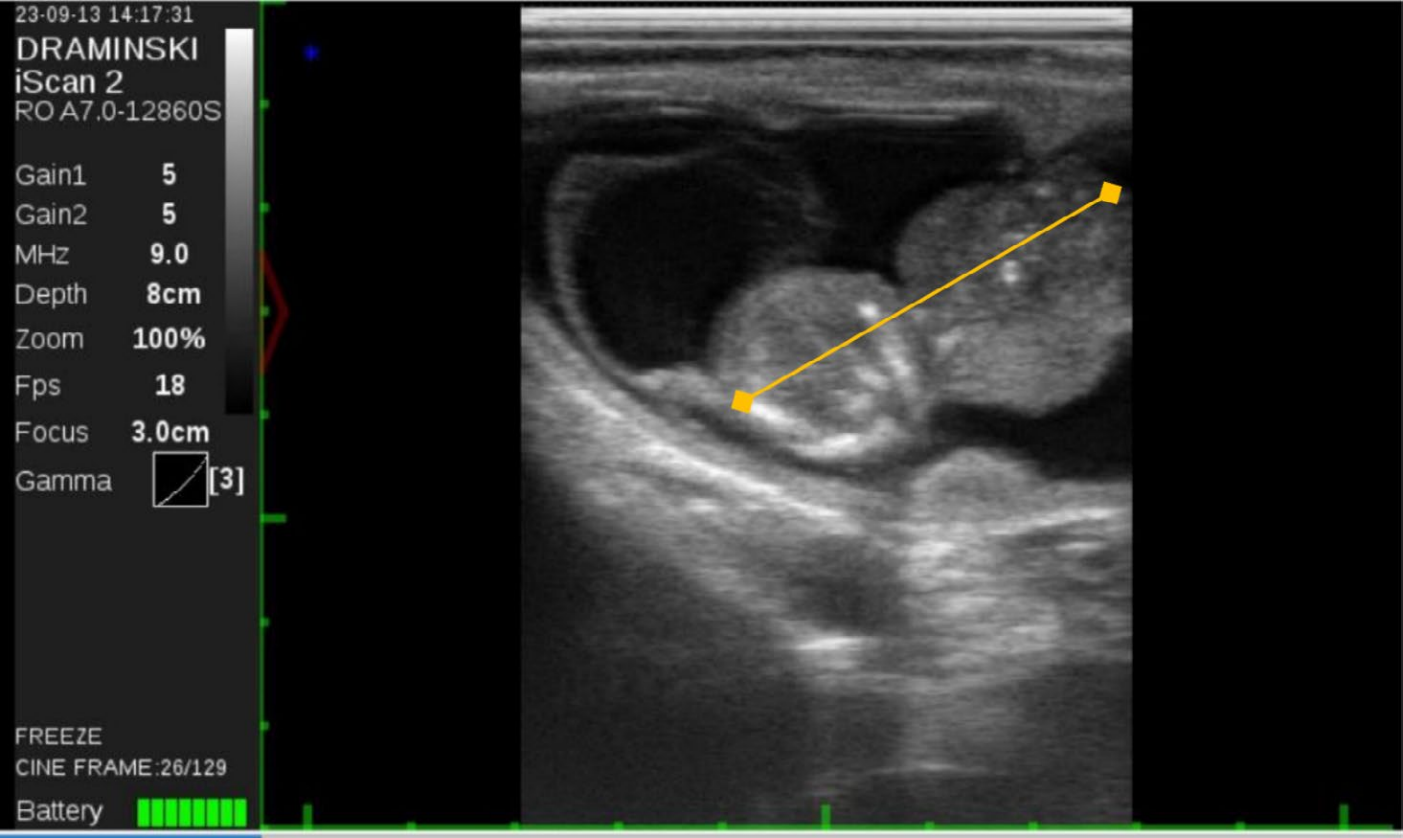
56‐day‐old foetus with a crown‐rump length (CRL) of 40.8 mm (orange line). The normal value in a healthy well‐formed foetus at 56 days of pregnancy is 50 mm.

**FIGURE 2:**
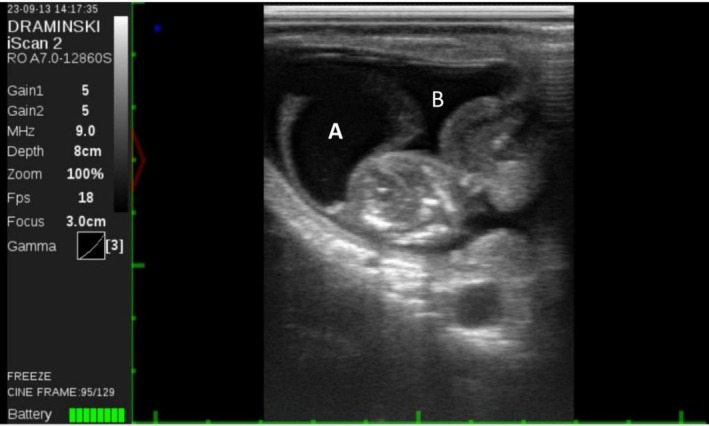
56‐day‐old foetus located outside the amniotic cavity (A) but still within the allantoic cavity (B). The foetus is presented as grossly malformed.

**FIGURE 3:**
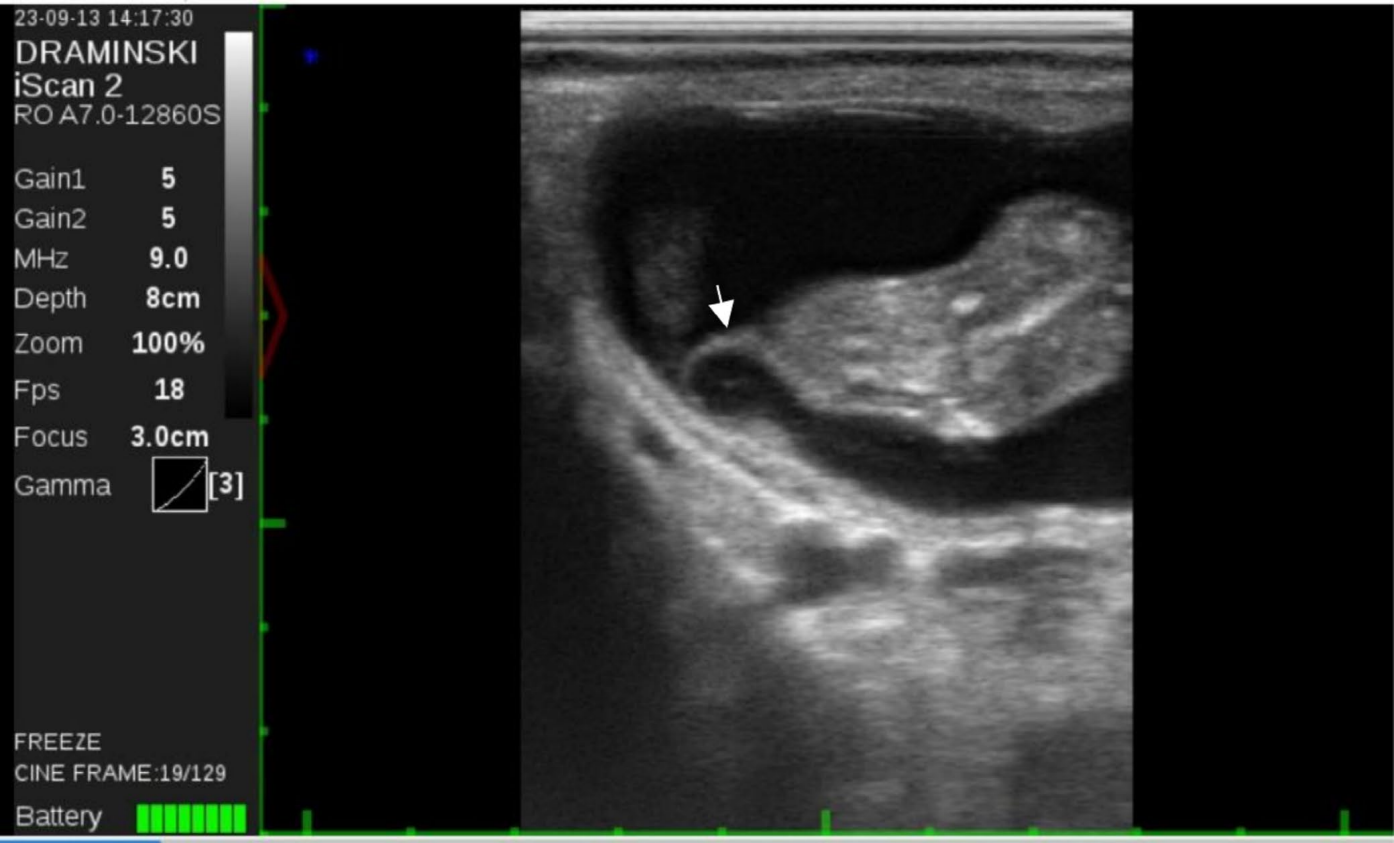
56‐day‐old foetus with a band‐like structure (arrow) attached to it. These structures can be suggestive of an amniotic band syndrome diagnosis.

The animal was then subjected to transrectal B‐mode ultrasound evaluation (Draminski, Poland) with a transrectal linear probe to visualise the contents of the uterus. The right‐horn pregnancy and the presence of an ipsilateral corpus luteum were confirmed. The foetus was identified but appeared extremely deformed caudally from the neck. There was a thorax‐like structure continuous with the neck, yet there was no evidence of the posterior part of the body or any of the limbs. There was a rhythmic movement compatible with a heartbeat and the foetus was considered alive. Foetal length was 41 mm, but the expected length of a 56‐day bovine foetus is 50 mm, according to the ultrasound software (Figure 1). Upon further examination, the foetus was located outside the amniotic sac (Figure 2), with band‐like structures attached to it (Figure 3). Abortion was induced with 0.15 mg of dexcloprostenol. It was not possible to recover the aborted material; most likely it was cleared by the automatic manure scraper after being expelled. Distinction between intrauterine amputation or aplasia of body parts was not possible. There was no evidence of an amniotic rupture.

The animal had no history of disease or treatment prior to this examination, and the milk progesterone concentration measured by the automatic milking system did not drop during the pregnancy.

After one oestrous cycle of rest, the cow was inseminated again, resulting in a normal pregnancy and an eutocic calving.

## 4 Discussion

In cattle, large congenital malformations can lead to abortions or severe dystocia, involving additional costs for the farm. Birth assistance was required in more than half of the cases of congenital abnormalities (107/191 = 56%; Mee et al. 2023). However, in a more recent study (Quigley and Mee 2025), it was reported that most congenital defects did not require calving assistance (243/327 = 74%). It is notable that the first study was based on veterinarian reports, while the second one was mainly from herdowner's reports. Veterinarians are not present at all calvings, and as such, that bias of cases seen may be behind the difference in results.

This case of an extra‐amniotic foetus with large malformations and the presence of band‐like structures is suggestive of an amniotic band syndrome diagnosis. While this syndrome has been reported in the cat (Martín‐Alguacil et al. 2025), the pig (Martín‐Alguacil and Avedillo 2019), and the rhesus monkey (Tarantal and Hendrickx 1987), it has not been described in cattle before, and it could not be confirmed in this case since it was not possible to recover the aborted material. Baca et al. (2009) documented a case of an extra‐amniotic pregnancy with foetal deformities that did not involve amniotic bands, which is also a possibility for the present case. Additionally, the possibility of this occurring due to severe dysplasia of the fetoplacental unit cannot be discarded.

It would be of interest to further examine calves born with large congenital defects to differentiate true aplasia from intrauterine amputations. The presence of amniotic membrane surrounding amputated or malformed organs could support the idea of the amniotic band syndrome existing in the bovine species. It is likely that the prognosis would be similar to what is seen in humans, varying greatly with the concomitant findings and not expected to reoccur in following pregnancies (Gandhi et al. 2019).

Extra‐amniotic foetal development was reported in this case; however, it is unknown whether the development would have continued and resulted in the birth of a healthy calf, as has been observed in humans (Kohler and Jenkins 1976). Nonetheless, in our case, there were additional large body defects that justified the interruption of the pregnancy.

Our case illustrates the importance of performing routine ultrasound early for pregnancy diagnosis for the premature identification of congenital malformations. This allows timely reaction for the prevention of calving problems, reducing economic losses.

## Author Contributions

Luís F.M.M. Esteves and Juhani Taponen were present and participated on the diagnosis of the malformation. Luís F.M.M. Esteves drafted the Manuscript. Juhani Taponen reviewd and approved its submission.

## Conflicts of Interest

The authors declare no conflicts of interest.

## Data Availability

Data sharing not applicable to this article as no datasets were generated or analysed during the current study.
